# The Impact of UV Radiation on *Paramecium* Populations from Alpine Lakes

**DOI:** 10.1111/jeu.12463

**Published:** 2017-09-08

**Authors:** Barbara Kammerlander, Barbara Tartarotti, Bettina Sonntag

**Affiliations:** ^1^ Lake and Glacier Research Group Institute of Ecology, University of Innsbruck Technikerstrasse 25 Innsbruck 6020 Austria; ^2^ Ciliate Ecology and Taxonomy Group Research Department for Limnology, Mondsee, University of Innsbruck Mondseestrasse 9 Mondsee 5310 Austria

**Keywords:** Ciliates, comet assay, DNA damage, protists, single‐cell gel electrophoresis

## Abstract

*Paramecium* populations from a clear and a glacier‐fed turbid alpine lake were exposed to solar simulated ultraviolet (UVR) and photosynthetically active radiation (PAR) at 8 and 15 °C. The ciliates were tested for DNA damage (comet assay), behavioral changes, and mortality after UVR + PAR exposure. High DNA damage levels (~58% tail DNA) and abnormal swimming behavior were observed, although no significant changes in cell numbers were found irrespective of the lake origin (clear, turbid), and temperatures. We conclude that environmental stressors such as UVR and their effects may influence the adaptation of ciliates living in alpine lakes.

ALPINE regions are faced with increasing temperatures (Vaughan et al. 2013) and the accelerated process of glacial melting due to climate warming will eventually turn turbid glacier‐fed lakes into clear ones when the connectivity to the glacier is lost (Sommaruga 2015). In contrast to highly turbid lakes, in clear alpine lakes UVR (280–400 nm) can penetrate the entire water column (Kammerlander et al. 2016; Sommaruga and Psenner 1997). The response to UVR of aquatic organisms originating from alpine lakes of contrasting UVR transparency was recently compared in copepods using a single‐cell gel electrophoresis method (comet assay; Tartarotti et al. 2014). Higher relative DNA damage accumulation under UVR exposure was observed in the population from the turbid lake. Adaptive traits such as photoprotection by sunscreen compounds and DNA repair mechanisms are seen to be a prerequisite for these organisms to thrive in clear lakes (Tartarotti et al. 2014).

However, nothing is known about the extent of UVR‐induced DNA damage in ciliated protists from alpine lakes. Ciliates are key organisms in microbial food webs, transferring energy from lower to higher trophic levels (Sommer et al. 2012). In alpine lakes, ciliate species richness and abundance are low and differ among clear and glacier‐fed turbid lakes (Kammerlander et al. 2016; Sonntag et al. 2011a; Wille et al. 1999). Apart from food (phytoplankton) and predatory zooplankton, the available underwater irradiance significantly influences the ciliate distribution and may structure the overall protistan community (Kammerlander et al. 2015, 2016). Generally, UVR has deleterious direct and indirect effects on organisms at the molecular and ecological level. UVR can cause single‐ and double strand breaks in the DNA and induce the synthesis of 6‐4 photoproducts and cyclobutane pyrimidine dimers, which may interfere with protein biosynthesis (e.g., Häder et al. 2015 and references therein). Under UVR exposure, the motility and cell division/reproduction rates of ciliates can be significantly affected (Giese 1945; Giese et al. 1963; Hörtnagl and Sommaruga 2007; Sgarbossa et al. 1995; Sommaruga et al. 1999). However, ciliate species respond individually to high UVR levels including avoidance (shading; Slaveykova et al. 2016), densely packed cell matter around the nuclei (algal symbionts; Sommaruga and Sonntag 2009; Summerer et al. 2009; Sonntag et al. 2011b), the acquisition of sunscreen compounds (mycosporine‐like amino acids; Sonntag et al. 2007, 2017), and/or effective DNA photorepair processes (Sanders et al. 2005). For example, photoenzymatic repair (PER) was reported in *Paramecium* (Sutherland et al. 1967; Takahashi et al. 2005; Zaar 1968) and experiments with *Glaucoma* and *Cyclidium* revealed that this mechanism was strongly temperature‐dependent and significantly more effective at higher temperatures (Sanders et al. 2005).

To shed light on UVR‐induced DNA damage and response (mortality and behavior) in ciliates from alpine lakes, we hypothesized that UVR‐induced DNA damage and mortality was higher in a *Paramecium* population from a less UV transparent glacially turbid lake than in a population from a clear lake. Under the assumption that less DNA damage and lower mortality occurred at a higher temperature due to the presence of possible temperature‐dependent repair mechanisms, both populations were cultivated and experimentally tested at 8 °C (i.e., mean lake temperature) and at 15 °C (i.e., close to the lake maximum temperature). To analyze the extent of DNA damage, we applied a modified alkaline comet assay that allows detecting and quantifying DNA damage of the macronucleus by measuring the migration of DNA from immobilized nuclear DNA (De Lapuente et al. 2015; Lee and Steinert 2003). Additionally, ciliate mortality and their swimming behavior were assessed before and after UVR exposure.

## Materials and Methods

### Sampling and cultivation

During summer 2010, we collected planktonic ciliate samples from a boat at the deepest point of the clear Gossenköllesee (GKS: 2,417 m a.s.l., max. depth: 9.9 m, area: 0.017 km²) and the glacier‐fed turbid Rifflsee (RIF: 2,234 m a.s.l., max. depth: 24 m, area: 0.269 km²; mean turbidity: 48.9 NTU, nephelometric turbidity units) by vertical net hauls (10‐μm mesh size). We also caught some individuals of *Paramecium* cf. *putrinum* in both lakes, which is a rare but cultivable ciliate species from these alpine sites. For cultivation, individual cells were cleaned with 0.2‐μm filtered lake water and grown in Woods Hole MBL Medium (WC medium) with an initial food concentration (*Cryptomonas* strain 26.80, algal culture collection Göttingen, Germany) of 7,767 ± 154 cells/ml. Cultures were kept in a climate chamber equipped with five Cool White lamps (Osram, Germany, L36/W20, emitting 180 μmol/m^2^/s PAR; 16:8 h light:dark cycle) and one A‐340 Q‐Panel lamp (Q‐Lab, Cleveland, OH, 290–380 nm, emitting 1.38 W/m^2^ UV‐B and 5.21 W/m^2^ UV‐A for 1 h/d) at two temperatures (8 and 15 °C). Growth rates (Table S1) were determined to identify the experimentally relevant late exponential growth phase (i.e. 19–21 d at 8 °C, 12–14 d at 15 °C).

### Experimental setup

All experiments were performed in a temperature‐controlled walk‐in chamber equipped with four A‐340 Q‐Panel lamps and two F36W/860 daylight lamps (General Electric Lighting, 400–700 nm). The lamps were placed 25 cm above the well‐plates. Two treatments and a control were exposed for 6 h to simulated natural irradiation conditions (spectrum available in Sommaruga et al. 1996): PAR only (well plates covered with Ultraphan‐395 foil, UV‐Opak, Digefra, Munich, Germany; sharp cut off: 0% transmittance at 390 nm, 50% at 405 nm), UVR + PAR, and a DARK control (well plates covered with aluminum foil).Two independent experiments were conducted each at 8 and 15 °C. In 12‐well culture plates, 400–600 ciliates per well were kept in WC medium (comet assay: 1 ml per well, mortality tests: 3.5 ml) with ~500 *Cryptomonas*/ml.

### Analysis of DNA damage

At the beginning of the experiments (*t*_0_) and after 6 h of exposure, six wells of the plate were pooled resulting in three replicates per treatment. Lah et al. (2004) introduced a comet assay protocol for ciliates, and since then mainly genotoxicity studies were conducted using different modifications (Hong et al. 2015; Kawamoto et al. 2010; Takada and Mastuoka 2009; Xu et al. 2008), indicating that the protocol needed to be adapted for every species. Our preliminary studies revealed that most of the *Paramecium* cells were not lysed and that high background DNA damage (80–90% tail DNA) occurred. Cell lysis was finally successful by immediately adding the cryoprotectant dimethyl sulfoxide (DMSO; Roth, Karlsruhe, Germany) at non‐toxic levels (< 10%) to avoid crystal formation (Azqueta and Collins 2013). The samples were frozen at −80 °C (30 min), thawed in a fridge at 8–10 °C for 30 min and placed in a water bath (room temperature, 5 min). To concentrate the cells, the samples were centrifuged (1.4 *g* × 1,000 for 1 min), and the supernatant removed. These steps resulted in a background DNA damage of 23.7 ± 11.8% tail DNA (comet tail length: 36.9 ± 12.4 μm; olive tail moment 6.1 ± 3.5) at *t*_0_, which coincides with previous studies (Kawamoto et al. 2010; Lah et al. 2004). All subsequent steps of the alkaline comet assay and the quantitation of the DNA damage followed a modified protocol of Tartarotti et al. (2014) and references therein. We applied a short lysis time (2 h) with a modified lysis buffer by adding Sarcosyl 0.2% (Sigma‐Aldrich, Vienna, Austria), and a short electrophoresis run (5 min). To prevent DNA damage caused by experimental handling, we kept the slides in the dark.

### Assessment of mortality and behavior

For mortality estimates, 0.5 ml of each replicate were preserved with 50 μl Lugol's solution at *t*_0_, 4, and 6 h, and cell numbers were estimated by direct counts (Olympus SZ 40, 100–400X magnification). The swimming behavior of the ciliates was recorded prior to preservation (Movies S1 and S2).

### Data analysis

To test for significant differences among treatments, the results from two experiments were summarized and the DNA damage (i.e. the relative percentage of DNA in the comet tail, % DNA in tail) was determined. The comet assay data were arcsin square root transformed and the data of the mortality tests were square root transformed. *T*‐tests and analyses of variance (ANOVA) were performed at a significance level of *P* < 0.05 (Bonferroni post hoc method; IBM SPSS Statistics 21.0, Armonk, NY, USA).

## Results and Discussion

**Figure 1:**
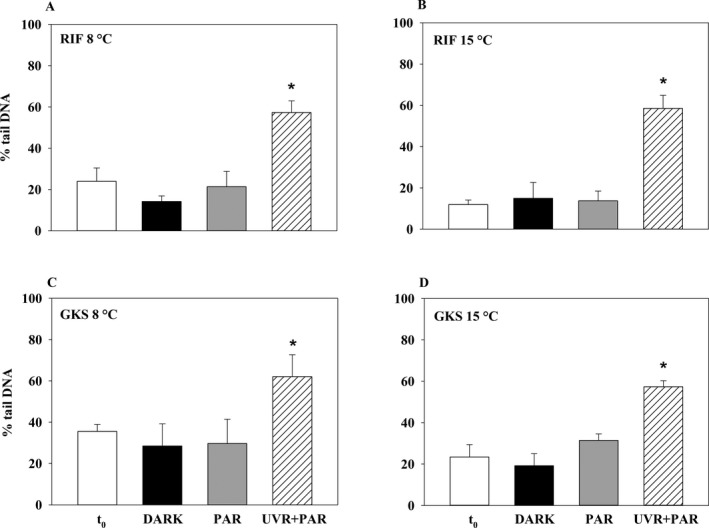
UVR‐induced DNA damage in *Paramecium* populations from one glacier‐fed turbid (Rifflsee, RIF;** A, B**) and one clear (Gossenköllesee, GKS;** C, D**) alpine lake cultivated and tested at 8 and 15 °C, respectively. DNA damage of the ciliates at the beginning of the experiment (*t*_0_), after 6 h of exposure to UVR including photo‐reactivating PAR (UVR + PAR), PAR only (PAR; UVR excluded), and when kept in the dark (DARK). DNA damages are presented as mean % tail DNA + standard deviation (two independent experiments were summarized, *n* = 3–6). Asterisks (*) above the bars indicates significant differences among the treatments (ANOVA; all pairwise multiple comparison procedure, Bonferroni method, *P* < 0.05).

Compared to *t*_0_, PAR, and DARK, the DNA damage of the ciliates was significantly higher after exposure to UVR + PAR (Fig. 1; *P* < 0.05). Neither between habitats (clear and turbid lake) nor temperatures (8 and 15 °C) statistically significant differences were observed in the DNA damage levels of the UVR‐exposed ciliates (RIF and GKS at 8 °C: 57.3% and 62.1% mean tail DNA; at 15 °C: 58.5% and 57.3%; *P* > 0.05). The extent of the DNA damage in the ciliates after UVR + PAR exposure was similar to the DNA damage levels reported for UV‐exposed copepods from clear and turbid alpine lakes (Tartarotti et al. 2014). The background damage at t_0_ was significantly higher (*P* < 0.05) at 8 °C and lower in the *Paramecium* population from the turbid than from the clear lake (not statistically significant; Fig. 1). These results support the findings of Tartarotti et al. (2014) showing that aquatic organisms such as copepods or ciliates (this study) originating from environments with less UV stress have low background damage levels, resulting in higher relative DNA damage accumulation. The hypothesis that at higher temperature reduced DNA damage and mortality occurred, because DNA repair mechanisms were more activated, could not be supported by our results.

Only UVR‐exposed individuals showed an abnormal swimming behavior (slowdown, see Movies S1 and S2) and were highly sensitive to further handling. Hörtnagl and Sommaruga (2007) also observed an erratic swimming pattern in the aposymbiotic congener *Paramecium bursaria* after UVR + PAR exposure. Abnormal swimming behavior was probably a consequence of damaged DNA strands in the macronucleus, which is regarded as the transcriptionally active center for physiological processes (Simon and Plattner 2015). In nature, limited motility and reduced speed velocity may increase the risk of predation. However, the UVR‐induced DNA damage did not cause significant mortality (*P* > 0.05; data not shown) and even one week later, the *Paramecium* were still alive (Kammerlander, pers. observ.).

Nevertheless, we cannot exclude long‐term effects such as retarded cell division (Giese 1945; Giese et al. 1963). This was only a short‐time experiment and an extended exposure to UVR might have more detrimental effects. Nuclear dimorphism may act as an “environmental stress buffer”, where possible severe effects to the diploid micronucleus may be buffered by the polyploid character of the macronucleus (Sperling 2011), as not all copies of a gene are probably affected by UVR. This is speculative, but the role of such buffering effects is still unclear and needs further genomic analyses.

In conclusion, to inhabit clear lakes implies the need of UVR tolerance/resistance and/or avoidance. Recently, we showed that some planktonic ciliate species are quite abundant in both clear and turbid alpine lakes, while other mainly particle‐associated species are only present in turbid habitats (Kammerlander et al. 2016). Here, we found *Paramecium* cf. *putrinum* in both lake types (clear and turbid) and by exposing them to UVR caused similar DNA damage and swimming deficiencies, but also a certain degree of survival and tolerance. Finding *Paramecium* in the pelagial is rare as these particle‐associated ciliates are typically colonizing benthic environments and detritus (Foissner et al. 1994). Their occurrence in the turbid lake may be related to suspended particles, whereas in the clear lake it appears likely that they were introduced into the pelagial by “wash out” processes from the littoral. Our results did, however, not support the initial hypothesis that UVR responses in *Paramecium* cf. *putrinum* depended on the habitat type (turbid vs. clear). Further experiments and (epi)genetic analyses are needed to shed more light on the potential role of UVR in influencing the occurrence of ciliated protists in alpine lakes.

## Supporting information


**Table S1.** Mean growth rate, mean doubling time, and time of the late exponential growth phase in days of the two *Paramecium* populations from the glacier‐fed turbid lake Rifflsee (RIF) and the clear lake Gossenköllesee (GKS) cultivated at 8 and 15 °C, respectively.

**Movie S1.** Swimming behavior of *Paramecium* after 6 h of exposure to UVR including photo‐reactivating PAR (UVR + PAR).

**Movie S2.** Swimming behavior of *Paramecium* after 6 h of exposure to PAR only (UVR excluded) and when kept in the dark (DARK), respectively.
