# Supplementary material for: The Impact of UV Radiation on Paramecium Populations from Alpine Lakes
Source: J Eukaryot Microbiol. 2017 Sep 8;65(2):250–4. doi: 10.1111/jeu.12463 (PMC5888136; doi:10.1111/jeu.12463)
Supplement: Supplementary file 1 — Table S1. Mean growth rate, mean doubling time, and time of the late exponential growth phase in days of the two Paramecium populations from the glacier‐fed turbid lake Rifflsee (RIF) and the clear lake Gossenköllesee (GKS) cultivated at 8 and 15 °C, respectively. [file JEU-65-250-s001.pdf]

SUPPORTING INFORMATION

The Impact of UV Radiation on *Paramecium* Populations from Alpine Lakes by Barbara Kammerlander, Barbara Tartarotti and Bettina Sonntag

Table S1. Mean growth rate, mean doubling time, and time of the late exponential growth phase in days of the two *Paramecium* populations from the glacier-fed turbid lake Rifflsee (RIF) and the clear lake Gossenköllesee (GKS) cultivated at 8 °C and 15 °C, respectively.

Movie S1. Swimming behavior of *Paramecium* after 6 h of exposure to UVR including photo-reactivating PAR (UVR + PAR).

Movie S2. Swimming behavior of *Paramecium* after 6 h of exposure to PAR only (UVR excluded) and when kept in the dark (DARK), respectively.

Table S1. Mean growth rate, mean doubling time, and time of late exponential growth phase in days of the two *Paramecium* populations from the glacier-fed turbid lake Rifflsee (RIF) and the clear lake Gossenköllesee (GKS) cultivated at 8 °C and 15 °C, respectively. Three independent experiments were summarized and the data presented as mean ± standard deviation.

Lake	Temperature (°C)	Mean growth rate (d^{-1})	Mean doubling time (d^{-1})	Time of late exponential growth phase (d)
**RIF**	**8**	2.92 ± 0.79	0.29 ± 0.06	21 ± 5
	**15**	3.78 ± 0.60	0.19 ± 0.03	17 ± 4
**GKS**	**8**	2.49 ± 0.20	0.28 ± 0.02	19 ± 3
	**15**	3.79 ± 0.39	0.18 ± 0.02	13 ± 1
